# *EGFR*外显子20插入突变阳性NSCLC治疗的临床研究进展

**DOI:** 10.3779/j.issn.1009-3419.2022.103.01

**Published:** 2022-05-20

**Authors:** 雪 杨, 军 赵

**Affiliations:** 100142 北京，北京大学肿瘤医院胸部肿瘤内一科 Department of Thoracic Medical Oncology, Peking University School of Oncology, Beijing Cancer Hospital & Institute, Beijing 100142, China

**Keywords:** 肺肿瘤, 表皮生长因子受体, EGFR外显子20插入突变, 靶向治疗, Lung neoplasms, Epidermal growth factor receptor, *EGFR* exon 20 insertion mutations, Targeted treatment

## Abstract

表皮生长因子受体（epidermal growth factor receptor, EGFR）外显子20插入突变是*EGFR*突变的第三大常见类别，约占所有*EGFR*突变阳性非小细胞肺癌（non-small cell lung cancer, NSCLC）的5%-12%。携带*EGFR*外显子20插入突变的NSCLC患者临床特征与“经典”*EGFR*激活突变人群相似，但预后很差。*EGFR*外显子20插入突变异质性高，可导致EGFR不同构象变化。大多数（几乎90%的病例）发生在*α*-C-螺旋后的Loop环结构区，最常报告的插入突变亚型为D770_N771 > ASVDN（A767_V769dupASV），突变频率为21%-28%。目前已知*EGFR*外显子20插入突变的NSCLC对既往已批准的EGFR酪氨酸激酶抑制剂原发性耐药，而且对传统化疗和免疫治疗也不敏感。近期Amivantamab与Mobocertinib在美国的获批改变了*EGFR*外显子20插入突变的NSCLC患者的治疗模式。此外，针对NSCLC *EGFR*外显子20插入突变的其他新型靶向药物正在研发中，期待为该类患者带来更多生存获益。本文就近年来伴有*EGFR*外显子20插入突变的NSCLC的临床研究进展进行归纳总结，旨在为该类患者的临床治疗提供参考。

## Abstract (en)

Epidermal growth factor receptor (*EGFR*) exon 20 insertion mutations are the third most prevalent activating *EGFR* mutation in non-small cell lung cancer (NSCLC), accounting for 5%-12% of all *EGFR* mutations in NSCLC cases. Patients harboring *EGFR* exon 20 insertion mutations exhibit similar clinical characteristics except for worse prognosis as compared to those with 'classic' *EGFR* mutations. *EGFR* exon 20 insertion mutations are considered as a heterogeneous class of alterations that cause different conformational changes in EGFR. The majority of mutations (almost 90% of cases) is positioned in the loop that immediately follows the C-terminal of the C-helix, and the most widely reported subtype of insertion mutations is D770_N771 > ASVDN(A767_V769dupASV) with frequency of 21%-28%. NSCLC patients with *EGFR* exon 20 insertion mutations show primary drug resistance to previously approved EGFR tyrosine kinase inhibitors and are generally insensitive to conventional chemotherapy and immunotherapy. The recently approved targeted drugs Amivantamab and Mobocertinib shift the treatment paradigm for NSCLC patients harboring *EGFR* exon 20 insertion mutations. There are also several new compounds targeting NSCLC *EGFR* exon 20 insertion mutations are in development. In this article, we provide a through overview on the treatment development in *EGFR* exon 20 insertion mutant NSCLC.

## 1 引言

### 1.1 表皮生长因子受体（epidermal growth factor receptor, *EGFR*）外显子20插入突变阳性非小细胞肺癌（non-small cell lung cancer, NSCLC）分子流行病学

*EGFR*外显子20插入突变占所有NSCLC腺癌突变的约2%，该突变频率在中西方NSCLC人群中相当^[1-6]^。继外显子19缺失和外显子21 L858R点突变（即两种“经典”*EGFR*激活突变），*EGFR*外显子20插入为第三种常见的*EGFR*突变类别，约占所有携带*EGFR*突变的NSCLC病例的5%-12%，该突变频率在中国*EGFR*突变阳性NSCLC人群中的报告结果低于西方人群（4.8%-5.1% *vs* 9%-12%）^[1-4, 6]^。与野生型或其他*EGFR*突变相比，*EGFR*外显子20插入突变更常见于女性、亚裔、非吸烟者、年长者以及NSCLC腺癌亚型，与*EGFR*常见突变临床特征相似^[7]^。在国内外指南中，*EGFR*外显子20插入突变已作为独立的分子分型进行治疗选择^[8, 9]^。

*EGFR*外显子20插入突变以*α*-C-螺旋附近密码子761与775之间框内插入和（或）复制为特征，进而导致*EGFR*通路构象性激活^[10, 11]^。就插入位点与氨基酸序列而言，*EGFR*外显子20插入突变异质性高，少部分位于*α*-C-螺旋的C末端（761-766），大多数（几乎90%的病例）发生在*α*-C-螺旋后的Loop环结构区（图 1）^[11]^。而且，*EGFR*外显子20 Loop环结构区内插入突变可进一步分为：Loop环近端（767-772）与Loop环远端（773-775），占比分别为66%-72%、27%-28%^[12]^。前者代表性突变为S768dupSVD、A767dupASV、D770insNPG以及D770delinsGY，后者包括H773insNPH、H773dupH、V774insAV以及V774insPR。根据几项大样本量（肿瘤或液体活检样本9142-24468例）分析^[3, 5, 6]^，最常报告的插入突变亚型为D770_N771 > ASVDN（A767_V769dupASV），突变频率21%-28%（图 2）。

**图 1:**
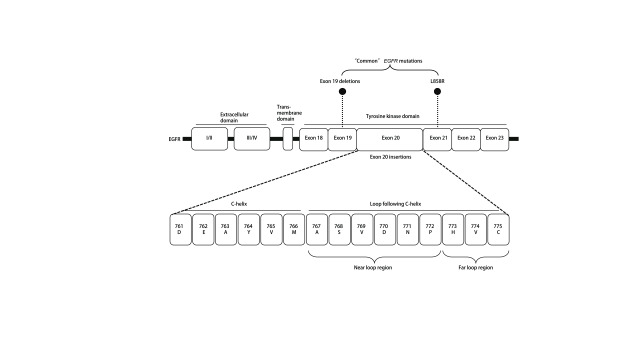
*EGFR*外显子20插入突变位置^[11]^。 Insertion site of *EGFR* exon 20 insertion mutants^[11]^. EGFR: epidermal growth factor receptor.

注：本图得到版权所有者©2020 Elsevier Ltd复制许可。

Note: Reprinted with the permission from the copyright holder ©2020 Elsevier Ltd.

**图 2:**
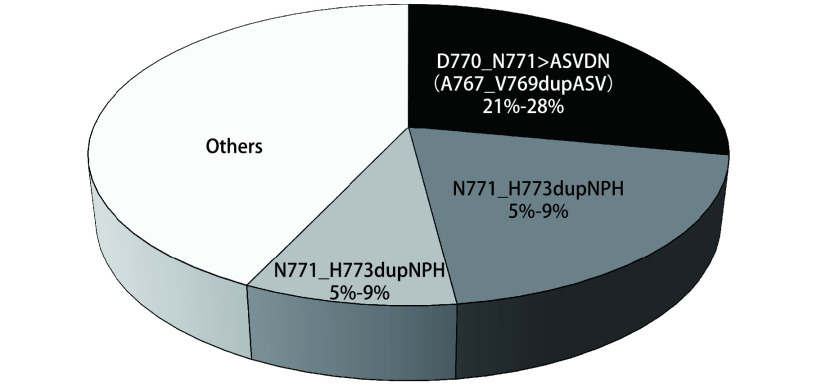
常见*EGFR*外显子20插入突变类型的发生频率^[3, 5, 6]^ Frequency of common *EGFR* exon 20 insertion mutations^[3, 5, 6]^

### 1.2 *EGFR*外显子20插入突变的异质性

目前已知*EGFR*外显子20插入突变的NSCLC对既往已批准的EGFR酪氨酸激酶抑制剂（tyrosine kinase inhibitor, TKI）原发性耐药（除A763_Y764insFQEA和A763_Y764insLQEA之外），而这种TKI耐药机制是多因素的^[8]^。*EGFR*外显子20插入突变诱导的刚性构象导致显著的空间位阻形成以及药物结合口袋变得小巧紧凑，进而阻止EGFR-TKI的有效结合^[13]^。不同于“经典”*EGFR*突变，*EGFR*外显子20插入突变通常能够在不高于ATP与野生型激酶亲和力的情况下激活*EGFR*^[10]^，使得ATP竞争抑制剂失去了靶向突变体而非野生型*EGFR*的选择性优势。这成为靶向*EGFR*外显子20插入突变的主要挑战。

除了插入亚型的多样性，*EGFR*外显子20插入突变对既往批准的EGFR-TKI的治疗反应也存在异质性。*α*-C-螺旋内（氨基酸761-766）的一些*EGFR*外显子20插入亚型对已获批的EGFR-TKI敏感（如A763_Y764insFQEA）^[10]^，而Loop环区内插入突变则治疗反应较差。此外，多项研究^[14-16]^表明，Loop环近端结构区内的插入突变类型的缓解率高于Loop环远端插入类型，*P*值分别为0.002, 5及0.027，但样本量相对较小。这些数据进一步提示*EGFR*外显子20不同结构域的插入类型对治疗方案的敏感性也存在差异，这些特征为*EGFR*外显子20插入突变NSCLC的临床治疗提供了思路。

### 1.3 *EGFR*外显子20插入突变检测

除了了解*EGFR*外显子20插入的分子流行病学特征及其不同亚型治疗反应的异质性之外，基因检测也是NSCLC治疗管理的关键环节，确切识别特异性序列亚型尤为重要。最常用的检测手段包括聚合酶链反应（polymerase chain reaction, PCR）和下一代测序（next-generation sequencing, NGS）等^[8]^。然而，PCR易漏检，需要相对较大的组织样本，通常用于识别最常见的遗传变异^[17]^，在*EGFR*外显子20插入突变等分子异质性突变检测方面能力不足^[3, 18, 19]^。NGS作为识别分子异质性序列改变的诊断工具，不但很快得出检测结果，而且使用单个组织样本即能够同时进行数千种基因检测，目前已检出NSCLC *EGFR*外显子20插入突变40种-102种亚型，敏感性更高，可靠性更强，因而更具价值^[17, 19]^。研究^[19]^显示，NGS识别的*EGFR*外显子20插入突变中有48.6%是PCR无法识别的。在美国，2011年-2019年临床上*EGFR*外显子20插入突变测序技术分析显示，在*EGFR*外显子20插入突变阳性的NSCLC中，采用PCR检测的比例从67%降至16%，NGS检测比例从0%升至64%^[20]^。上述检测手段的转变提高了NSCLC中*EGFR*外显子20插入突变人群的检出率，从而使得精准治疗成为可能。中国NSCLC患者数量庞大，更需要全面而准确的肿瘤基因诊断手段。《二代测序技术在NSCLC中的临床应用中国专家共识（2020版）》建议，应优先使用国家药品监督管理局（National Medical Products Administration, NMPA）批准的传统或NGS检测产品，在NMPA批准的检测产品无法满足临床需求的情况下，可以考虑使用美国食品药品监督管理局（Food and Drug Administration, FDA）批准的NGS检测产品^[21]^。尽管作为首选的推荐检测手段，临床实践上NGS在中国*EGFR*外显子20插入突变阳性的NSCLC患者中的应用情况仍不清楚。

## 2 化疗、免疫肿瘤治疗及传统EGFR-TKI在*EGFR*外显子20插入突变NSCLC中的研究与探索

在Amivantamab（JNJ-6372）与Mobocertinib（TAK-788）获得FDA批准之前，携带*EGFR*外显子20插入突变的NSCLC患者最常用的治疗方案为含铂化疗等方案，其次为传统EGFR-TKI和免疫肿瘤治疗。一直以来，上述治疗手段因敏感性低、疗效差等，无法满足这类人群的临床需求。

### 2.1 化疗和免疫肿瘤治疗

据美国癌症电子病历Flatiron Health数据库截至2020年2月记录^[22]^，化疗及化疗联合方案是*EGFR*外显子20插入突变阳性NSCLC最常用的一线治疗，约占全部治疗手段的60%，获得的总缓解率（overall response rate, ORR）接近20%（*n*=57），中位无疾病进展生存期（progression-free survival, PFS）为4.5个月-5.7个月。免疫肿瘤治疗作为单药一线方案时，确认的ORR仅为9.1%，PFS为3.1个月（*n*=11）；作为铂类经治后≥二线治疗时，ORR为5.0%，PFS为2.2个月（*n*=20）。传统EGFR-TKI作为单药一线方案时，ORR仅为2.7%，PFS为3.3个月（*n*=37）；作为铂类经治后≥二线治疗时，ORR为10.0%，PFS为3.4个月（*n*=10）^[22]^。

### 2.2 传统EGFR-TKI

根据Flatiron Health数据库2011年1月-2020年5月记录^[23]^，与携带“经典”*EGFR*突变相比，传统EGFR-TKI在*EGFR*外显子20插入突变人群中的疗效不理想。新确诊*EGFR*外显子20插入突变阳性的转移性NSCLC患者真实世界中位总生存期（overall survival, OS）为16.2个月，低于*EGFR*外显子19缺失/L858R突变的25.5个月^[23]^。一代/二代EGFR-TKI厄洛替尼、吉非替尼、阿法替尼一线治疗*EGFR*外显子20插入突变阳性晚期NSCLC的小样本研究^[24]^显示，中位PFS < 3个月。ECOG-ACRIN 5162研究评估了三代EGFR-TKI奥希替尼160 mg在*EGFR*外显子20插入突变阳性晚期NSCLC中作为≥二线治疗的结果。ORR为25%[5/20，包含1例完全缓解（complete response, CR）]，疾病控制率（disease control rate, DCR）85%（17/20），中位PFS为9.7个月^[25]^（表 1）。该结果在随后的POSITION20研究^[26]^中得到证实。奥希替尼160 mg获得的ORR为28%（7/25），中位PFS为6.8个月，中位OS为15.2个月。然而，韩国（*n*=15）和日本（*n*=12）的小规模单臂研究^[27, 28]^均显示，标准剂量奥希替尼80 mg未能为*EGFR*外显子20插入突变阳性NSCLC患者带来临床缓解（ORR为0）。应在更大规模人群中深入探索。

**表 1:** 针对*EGFR*外显子20插入突变阳性NSCLC的药物临床数据汇总^[14, 15, 25-28, 40, 41, 44, 47, 50]^ Summary of clinical data of treatment for *EGFR* exon 20 insertion mutation positive NSCLC^[14, 15, 25-28, 40, 41, 44, 47, 50]^

Drug	MOA	Manufacturer	Study	EGFR ex20ins mutation positive NSCLC included	No. of pts	ORR (n/N)	mPFS (mon)	Safety profiles
Osimertinib^[25-28]^ (EGFR-TKI)	Binds irreversibly to certain mutant forms of *EGFR* (T790M, L858R, and exon 19 deletions) at approximately 9-fold lower concentrations than wild-type	AstraZeneca	ECOG-ACRIN 5162 (phase 2) Osimertinib 160 mg	At least one prior line of therapy was required; stable, asymptomatic brain metastases were allowed	20	25% (5/20)	9.7	The majority was grade 1-2. The most common AEs of any grade included diarrhea (76%), fatigue (67%), thrombocytopenia (67%); one case of grade 4 respiratory failure. One patient discontinued study treatment due to grade 3 anemia
			POSITION20 (phase 2) Osimertinib 160 mg	EGFR exon 20 positive (mutation, deletion and/or insertion); pre-treatment chemotherapy allowed; asymptomatic brain metastasis	25	28% (7/25)	6.8	The majority was grade 1-2. The most common TRAEs of any grade included diarrhea (72%), dry skin (44%), and fatigue (44%). Two pts discontinued study treatment due to grade 3 pneumonitis (*n*=1) and left ventricular systolic dysfunction (*n*=1). 5 pts had dose reduction from 160 mg to 80 mg
			AEX20 (phase 1/2) Osimertinib 80 mg	Previously treated with platinum containing chemotherapy	12	0% (0/12)	NR	NR
			LU17-19 (phase 2) Osimertinib 80 mg	Disease progression following or intolerant to adequate standard treatment; stable brain metastases	15	0% (0/15)	3.5	The most common AEs of any grade included nausea (20%), vomiting (20%) and anemia (13.3%).
Mobocertinib^[15]^ (EGFR-TKI)	Targets the protein in vicinity of EGFR exon 20 *α*-C- helix to obtain selectivity; exert antitumor activity through substitution on the pyrimidine ring	Takeda	TAK-788 phase 1/2 study (NCT02716116)	Previously treated with platinum containing chemotherapy	114	28% (32/114, assessed by IRC) 35% (40/114, assessed by INV)	7.3	The most common TRAEs of any grade included diarrhea (91%), rash (45%) and paronychia (38%). TRAEs of ≥ grade 3 was 47%, AEs leading to reduction was 25%; AEs leading to treatment discontinuation was 17%
DZD9008^[14]^ (EGFR-TKI)	Designed for *EGFR* and *HER2* exon 20 insertion mutations	Dizal	WU KONG1/WU KONG2 (phase 1/2)	Disease progression following or intolerant to adequate standard treatment; stable brain metastases	56	39.3% (22/56）	NR	The most common TEAEs included diarrhea (grade 3, 5.2%) and rash (grade 3, 1%)
Poziotinib^[40, 41]^ (EGFR-TKI)	Compared to the first-generation EGFR-TKI, its smaller molecule size and greater structural flexibility allows it to circumvent the steric hindrance induced by ex20ins mutants and inhibit *EGFR* and *HER2* exon 20 mutation with potency	Hanmi	ZENITH20-1 (phase 2)	Metastatic; any previous number of lines for systematic or targeted therapy were allowed	115	ITT: 14.8% (17/115) Evaluable pts: 19.3% (17/88)	4.2	60% of pts experienced AEs of ≥ grade 3; the most common TRAEs included rash (27.5%) and diarrhea (12.5%). 45% of pts had dose reduced to 12 mg and 17.5% reduced to 8 mg
			ZENITH20-3 (phase 2)	Treatment naive	79	27.8% (22/79)	7.2	The incidence of ≥ grade 3 rash was 33%, and diarrhea was 23%
CLN-081^[44]^ (EGFR-TKI)	Has unique pyrrole-aminopyrimidine frame and strong broad-spectrum activity against *EGFR* mutations including exon 20 insertion mutation	Cullinan Oncology authorized by Taiho Pharmaceutical	NCT04036682 (phase 1/2a)	≥1 prior platinum-based chemotherapy; Brain metastases that are stable ≥ 4 weeks	45	Evaluable pts: 50% (21/42)	NR	The most common TRAEs included rash (76%), diarrhea (22%), and paronychia (22%). 44% of pts had grade 3 AE with liver injury
Amivantamab^[47]^ (bispecific EGFR-c-metantibody)	Disrupts EGFR and MET signaling functions through blocking ligand induced activation and degradation of EGFR and MET; guides immune effector cells targeting tumors harboring activated and drug-resistant *EGFR*/*MET* mutations and amplifications	JNJ/Janssen	CHRYSALIS (phase 1, cohort 4)	Previously treated with platinum containing chemotherapy	81	Assessed by IRC: 40% (32/81) Assessed by INV: 36% (29/81)	8.3	The most common TRAEs included rash (86%), IRRs (66%) and paronychia (45%). TRAEs ≥ grade 3 were 16%; Treatment-related dose reductions occurred in 13% of pts, dose interruption in 21%, and discontinuation in 4%. 94% of IRRs were primarily limited to the first infusion, and rarely occurred with further dosing. MET-related AEs were hypoalbuminemia (27%; grade 3 was 3%) and peripheral edema (18%; grade 3 was 0%)
Luminespib or AUY922^[50]^ (hsp90 inhibitor)	Leads to the degradation of client (including ex20ins mutation) proteins, by the ubiquitin proteasome pathway	Novatis	Single arm (phase 1)	≥1 prior therapy	29	17% (5/29)	2.8	The most common TRAEs were diarrhea (83%; grade 3 of 0%), visual changes (76%, grade 3 of 3%). 59% of pts required treatment delays or interruptions
AE: adverse event; DCR: disease control rate; ex20ins: exon 20 insertion; HER: human epidermal growth factor receptor; INV: investigator; IRC: independent review committee; IRR: infusion-related reaction; ITT: intent-to-treat; MET: mesenchymal epithelial transition factor; MOA: mechanism of action; NSCLC: non-small cell lung cancer; ORR: objective response rate; PFS: progression-free survival; pts: patients; TKI: tyrosine kinase inhibitor; TEAE: treatment-emergent adverse event; TRAE: treatment-related adverse event.								

此外，体内外研究^[29]^证实阿法替尼或奥希替尼联合西妥昔单抗对*EGFR*外显子20插入突变的有效性高于任一单药。少数病例报告显示阿法替尼^[30]^或奥希替尼^[31]^联合西妥昔单抗后线或二线治疗有一定疗效，但需要进一步临床研究证实EGFR-TKI+EGFR单抗的有效性。

## 3 *EGFR*外显子20插入突变阳性NSCLC治疗新策略

如前文所述，*EGFR*外显子20插入突变阳性NSCLC患者治疗面临着相当大的挑战，长期以来，化疗、EGFR-TKI、免疫肿瘤治疗等方案获益有限，患者生存结局差，亟需全新的靶向药物来填补该类人群未被满足的临床需求。

靶向药物Amivantamab和Mobocertinib先后于2020年3月与4月被美国FDA授予突破性治疗药物资格，用于既往接受含铂化疗时或之后疾病进展的*EGFR*外显子20插入突变阳性的局部晚期或转移性NSCLC成人患者。2021年5月与9月，Amivantamab和Mobocertinib先后通过美国FDA优先审评程序，获得该适应证加速批准，成为针对*EGFR*外显子20插入突变NSCLC的靶向药物。同时，上述两种药物均于2020年被NMPA纳入了“突破性治疗药物”程序。Mobocertinib已正式获得NMPA药品审评中心受理，并获准纳入“优先审评”审批程序，将有望成为国内首个获批上市的针对*EGFR*外显子20插入突变阳性患者治疗的口服靶向药物。目前已有成熟的有效性与安全性数据的临床研究汇总于表 1。

### 3.1 EGFR-TKI

#### 3.1.1 Mobocertinib

Mobocertinib是首个专门设计的选择性靶向*EGFR*外显子20插入突变的口服TKI，通过靶向*α*-C-螺旋附近的蛋白来获得选择性，通过在嘧啶环上的取代来发挥抗肿瘤活性，而奥希替尼不能利用该结合位点。Mobocertinib在嘧啶环上引入了异丙酯，能够与该空置口袋的守门残基相互作用。这种结构设计使得Mobocertinib对外显子20突变的亲和力强于奥希替尼，并且对外显子20激活突变的选择性和抑制力高于野生型*EGFR*^[32]^。

Mobocertinib的获批依据基于一项Ⅰ期/Ⅱ期临床研究（NCT02716116）（图 3）。将既往接受过含铂化疗的114例患者[即铂类经治人群（platinum-pretreated patient, PPP），包括剂量递增研究（*n*=6）、扩展队列（*n*=22）以及EXCLAIM延展队列（*n*=86）]进行合并分析，PPP人群的治疗方案为Mobocertinib 160 mg，每日1次（其中35%的患者基线伴脑转移，59%的患者既往接受过≥二线治疗，43%的患者既往接受过免疫治疗）^[15]^。主要研究终点显示，独立审查委员会（Independent Review Committee, IRC）评估确认的ORR为28%（32/114），研究者（investigator, INV）评估确认的ORR为35%（40/114）；IRC评估的中位缓解持续时间（duration of response, DOR）为17.5个月，INV评估的DOR为11.2个月；INV与IRC评估确认的DCR[≥部分缓解（partial response, PR）+疾病稳定（stable disease, SD）≥ 6周）]均为78%（89/114）；IRC评估确认的中位PFS为7.3个月，中位OS为24.0个月^[15]^。

**图 3:**
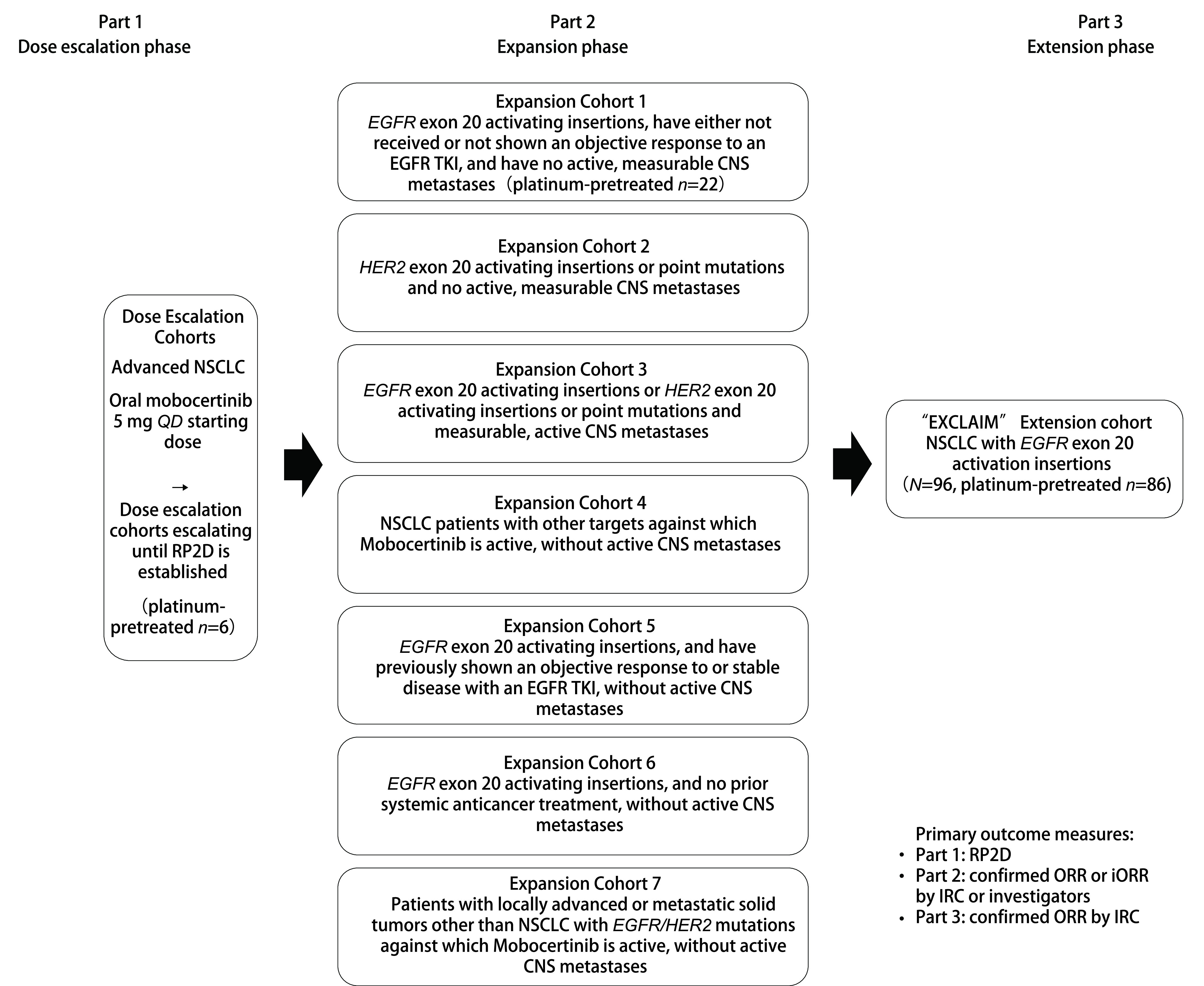
Mobocertinib在*EGFR*外显子20插入阳性NSCLC中的Ⅰ期/Ⅱ期研究设计^[15]^。 Design of mobocertinib phase 1/2 study in NSCLC patients with *EGFR* exon 20 insertions^[15]^. CNS: central nervous system; iORR: intracranial objective response rate; *QD*: daily; RP2D: recommended phase 2 dose.

注：本图得到版权所有者©2021 JAMA Oncology复制许可。

Note: Reprinted with the permission from the copyright holder ©2021 JAMA Oncology.

95例检出确切插入序列的患者中，发现 > 30种独立的*EGFR*外显子20插入突变类型，无论突变亚型频率还是距离C-螺旋区的位置如何，所有*EGFR*外显子20插入突变亚型均观察到缓解[ORR：ASV/SVD/NPH亚组31.9%（15/47）*vs*其他25.0%（12/48）；Loop环近端（767-772）28.6%（20/70）*vs* Loop环远端（773-775）25.0%（6/24）]（图 4）^[15]^。对于既往EGFR-TKI治疗时获得客观缓解或疾病稳定≥6个月后疾病进展的难治性*EGFR*外显子20插入突变人群（队列5；*n*=20），ORR为40%（基于IRC评估；8/20）或20%（基于INV评估；4/20），DCR为90%（基于IRC或INV评估），中位PFS为7.3个月（基于IRC评估），中位OS尚未达到^[33]^。对于EXCLAIM队列，治疗2个月后NSCLC核心症状改善，患者报告的整体健康相关生活质量在Mobocertinib治疗期间保持稳定^[34]^。

**图 4:**
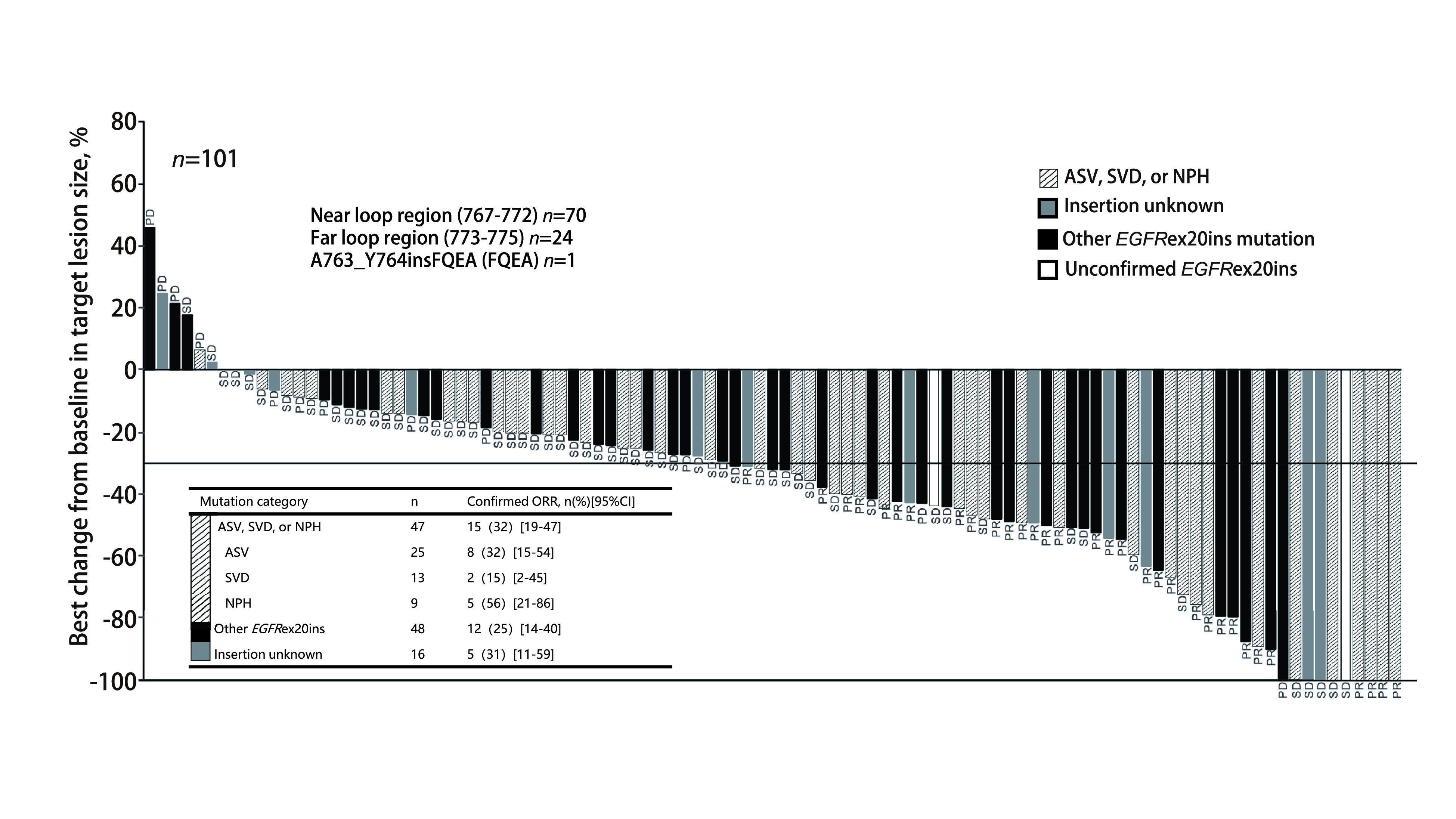
Mobocertinib在Ⅰ期/Ⅱ期研究PPP人群中对不同*EGFR*外显子20插入突变亚型的有效性^[15]^。 Objective response by *EGFR* exon 20 insertion mutation category (PPP cohort) in mobocertinib phase 1/2 study^[15]^. PD: progressed disease; PR: partial response; PPP: platinum-pretreated patient; SD: stable disease.

注：本图得到版权所有者© 2021 JAMA Oncology复制许可。

Note: Reprinted with the permission from the copyright holder © 2021 JAMA Oncology.

Mobocertinib安全性与已知的EGFR-TKI毒性反应特征一致，以胃肠道与皮肤黏膜不良事件（adverse event, AE）为特征。几乎全部患者出现治疗相关的任何AE，前三位分别为腹泻（91%）、皮疹（45%）、甲沟炎（38%）。治疗相关的任何≥3级AE发生率为47%，导致减量或停药的AE发生率分别为25%、17%。导致停药的最常见AE包括腹泻（4%）、恶心（4%）、呕吐（2%）、食欲减退（2%）以及口腔炎（2%）^[15]^。因AE而减量的患者ORR（21%, *n*=29）低于未曾因AE而减量的患者（31%, *n*=85），中位DOR（5.7个月*vs* 17.5个月）、中位PFS均更短（5.9个月*vs* 7.3个月）。这凸显了Mobocertinib治疗过程中早期识别和积极管理胃肠道与皮肤相关毒性反应的重要性^[35, 36]^。

Mobocertinib一线研究探索正在积极进行中。EXCLAIM-2（NCT04129502）随机化、开放性、国际多中心、Ⅲ期临床研究将比较Mobocertinib与含铂化疗（培美曲塞+顺铂或卡铂继以培美曲塞维持）一线治疗局部晚期或转移性*EGFR*外显子20阳性NSCLC的有效性及安全性。研究采取头对头设计，允许化疗组在疾病进展后交叉换组接受Mobocertinib。主要终点为IRC评估的PFS，关键次要终点为IRC评估的ORR以及OS。目前正在北美、欧洲以及亚太地区招募患者，目标样本量为318例^[37]^。

#### 3.1.2 DZD9008（Sunvozertinib）

DZD9008为针对*EGFR*/人类表皮生长因子受体2（human epidermal growth factor receptor-2, *HER*2）外显子20插入突变设计的选择性、不可逆EGFR-TKI。Ⅰ期/Ⅱ期临床研究（WU KONG 1和WU KONG 2）（NCT03974022和CTR20192097）汇总分析中，对于59例*EGFR*外显子20插入突变阳性患者，DZD9008 50 mg-400 mg均耐受性良好。剂量限制性毒性反应为腹泻与心律不齐。整体AE可管理，最常见的治疗中出现的AE为腹泻（3级，5.2%）和皮疹（3级，1%）^[14]^。56例患者有效性可评估，既往治疗线数中位值为2（1-10），基线期42.9%的患者伴脑转移。PR见于≥100 mg剂量水平所有队列。所有剂量组的ORR为39.3%（22/56）^[14]^。DZD9008推荐Ⅱ期剂量为300 mg每日1次，该队列ORR为48.4%（15/31），DCR为90.3%（28/31）；抗肿瘤活性见于不同突变亚型^[38]^。2020年12月，DZD9008被NMPA纳入“突破性”药物名单，拟用于治疗既往至少接受过一次全身化疗的携带*EGFR*外显子20插入突变的局部晚期或转移性NSCLC。

#### 3.1.3 波奇替尼

波奇替尼为新型第二代EGFR抑制剂，比第一代EGFR-TKI分子更小，结构上更灵活，可绕过外显子20插入突变诱导的空间位阻，强力抑制*EGFR*和*HER2*外显子20突变^[13]^。

Ⅱ期研究（NCT03066206）显示，40例疗效可评估的*EGFR*外显子20插入突变阳性NSCLC接受波奇替尼（16 mg，每日1次），其中65.1%的患者既往接受过针对转移性疾病的≥二线治疗。60%的患者出现≥3级AE，最常见的为皮疹（27.5%）和腹泻（12.5%）。45%的患者需要减量至12 mg，17.5%需减量至8 mg。8周时，ORR为58%（23/40），DCR为90%。中位PFS为5.6个月^[39]^。

然而，此后的多队列、多中心、Ⅱ期研究ZENITH20^[40]^中，队列1与3子研究均未达主要终点。ZENITH20-1入组115例*EGFR*外显子20插入突变阳性NSCLC，既往治疗中位线数为2。波奇替尼（16 mg，每日1次）获得的ORR为14.8%（17/115），DCR为68.7%，DOR为7.4个月，中位PFS为4.2个月。88例可评估患者中经过确认的ORR为19.3%（17/88），未经过确认的ORR为25%（22/88）。最常见的≥3级AE为皮疹（28%）、腹泻（26%）。ZENITH20-3入组79例*EGFR*外显子20插入突变阳性NSCLC初治患者，波奇替尼一线治疗（16 mg，每日1次）获得的ORR为27.8%（22/79），但95%置信区间下限（18.4%）低于预先设定的20%。91%的患者出现肿瘤缩小。中位PFS为7.2个月。≥3级皮疹发生率33%，腹泻为23%^[41]^。ZENITH20研究^[41, 42]^开放了队列5（*n*=40）以评估波奇替尼低剂量（16 mg *vs* 12 mg *vs* 10 mg）和新方案（每日2次取代每日1次）对经治或初治*EGFR*或*HER2*外显子20插入突变阳性NSCLC的耐受性，初步结果提示每日2次方案使皮疹、腹泻、口腔炎等AE发生率减少（*n*=122）。

ZENITH20-2研究^[43]^中波奇替尼二线治疗*HER2*外显子20插入突变NSCLC达到了有效性终点。目前已就该适应证获得FDA快速审批资格。而对于*EGFR*外显子20插入突变NSCLC，波奇替尼对初治患者有一定临床活性，每日2次减量方案可改善安全性，避免因耐受问题而带来的对完成用药周期的干预，需结合未来有效性数据来明确该方案的临床价值。

#### 3.1.4 CLN-081（TAS6417）

CLN-081是一种新型抗EGFR-TKI，对外显子20插入突变具有相对于野生型*EGFR*的高选择性。Ⅰ期/Ⅱa期研究（NCT04036682）在剂量递增阶段探索了CLN-081剂量水平30 mg、45 mg、65 mg、100 mg以及150 mg，每日2次。有效性扩展队列包括30 mg、65 mg、100 mg剂量组。在达到研究方案设定的安全性与有效性标准后，在100 mg剂量组启动Ⅱa期扩增研究^[44]^。主要终点为全部队列治疗中出现的AE发生率和严重程度、剂量限制性毒性反应发生率和严重程度、实验室结果异常发生率、生命指征异常发生率以及Ⅱa期剂量扩增队列中的ORR^[44]^。

截至中期数据分析时，入组45例*EGFR*外显子20插入突变NSCLC，既往均接受过≥一线含铂系统性化疗，73%的患者既往治疗线数≥2。45例安全性可评估患者中，最常见的药物相关AE为皮疹（76%）、腹泻（22%）、甲沟炎（22%）。44%的患者发生3级AE且伴肝损伤。CLN-081在42例疗效可评估患者中的总体ORR为50%（21/42），DCR为64%^[44]^。

### 3.2 双特异性抗体

Amivantamab为全人源EGFR-间质表皮转化因子（mesenchymal-epithelial transition, MET）双特异性抗体，与EGFR和MET的细胞外结构域相结合，具有多重抗癌机制。Amivantamab通过阻断配体诱导的激活以及诱导受体降解而抑制EGFR和MET信号通路，还引导免疫效应细胞靶向携带激活和耐药*EGFR*/*MET*突变与扩增的肿瘤^[45]^。由于EGFR与MET通路之间存在信号干扰，MET通路激活为EGFR-TKI耐药的重要的替代或旁路机制，对EGFR与MET的双重抑制使得Amivantamab能够在活性位点绕过对TKI的原发性与继发性耐药^[45, 46]^。

Amivantamab的获批基于多中心、开放性、多队列、Ⅰ期CHRYSALIS研究（NCT02609776）队列4的结果（图 5）。中位随访9.7个月时，共81例携带*EGFR*外显子20插入突变的晚期NSCLC成人患者纳入有效性分析。这些患者既往均在接受含铂化疗期间或之后疾病进展，40%为亚裔，22%基线期伴脑转移。既往治疗中位线数为2（1-7），25%的患者既往接受过EGFR-TKI治疗，46%接受过免疫治疗^[47]^。主要观察指标报告显示，对于有效性人群（*n*=81），基于盲法独立中心审查（blinded independent central review, BICR）评估的ORR为40%（32/81），CR为4%，PR为36%；INV评估的ORR为36%（29/81）。基于BICR评估的DOR为11.1个月，63%的患者DOR≥6个月。基于BICR的临床获益率（≥PR+SD≥11周）为74%，基于INV的临床获益率（≥PR+SD ≥11周）为73%^[47]^。

**图 5:**
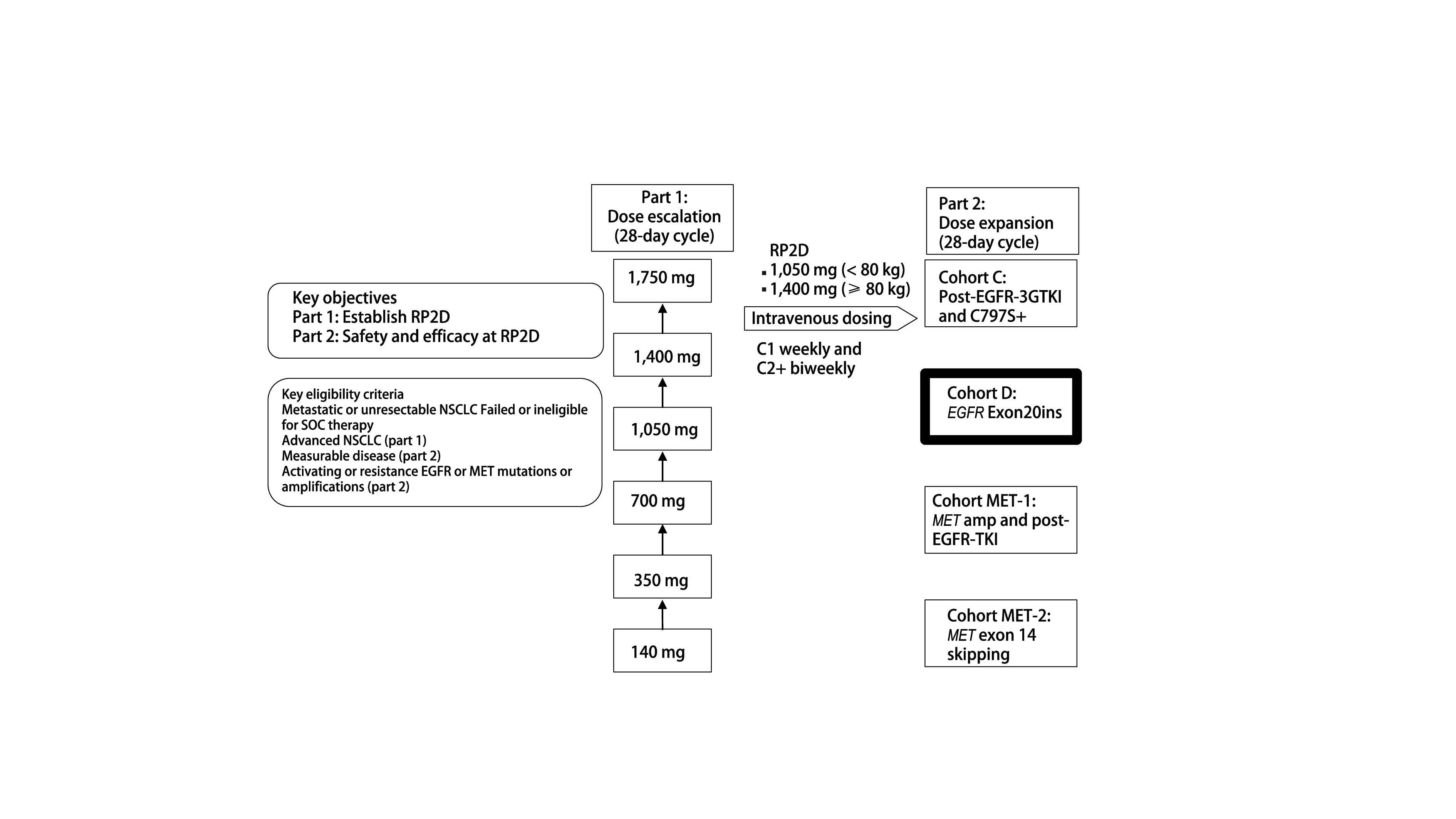
CHRYSALIS研究设计^[47]^。 Study design of CHRYSALIS^[47]^. C: cycle; SOC: standard of chemotherapy.

注：本图得到版权所有者© 2021 by American Society of Clinical Oncology复制许可。

Note: Reprinted with the permission from the copyright holder © 2021 by American Society of Clinical Oncology

基于BICR评估的抗肿瘤活性见于全部预先设定以及事后分析亚组，包括基线期是否伴脑转移[ORR: 39% (7/18) *vs* 40% (25/63)]，既往是否接受过免疫治疗[ORR: 46% (17/37) *vs* 34% (15/44)]、既往是否接受过EGFR-TKI [ORR: 50% (10/20) *vs* 36% (22/61)]^[47]^。全部81例患者均进行了ctDNA或肿瘤样本中心实验室检测，其中63例ctDNA可检出，共发现25种外显子20插入突变亚型。无论插入发生在*α*-C-螺旋区[ORR为100%（1/1）]、Loop环近端[ORR为41%（22/54）]或Loop环远端结构区[ORR为25%（2/8）]，均观察到不同程度的缓解（图 6）^[47]^。

**图 6:**
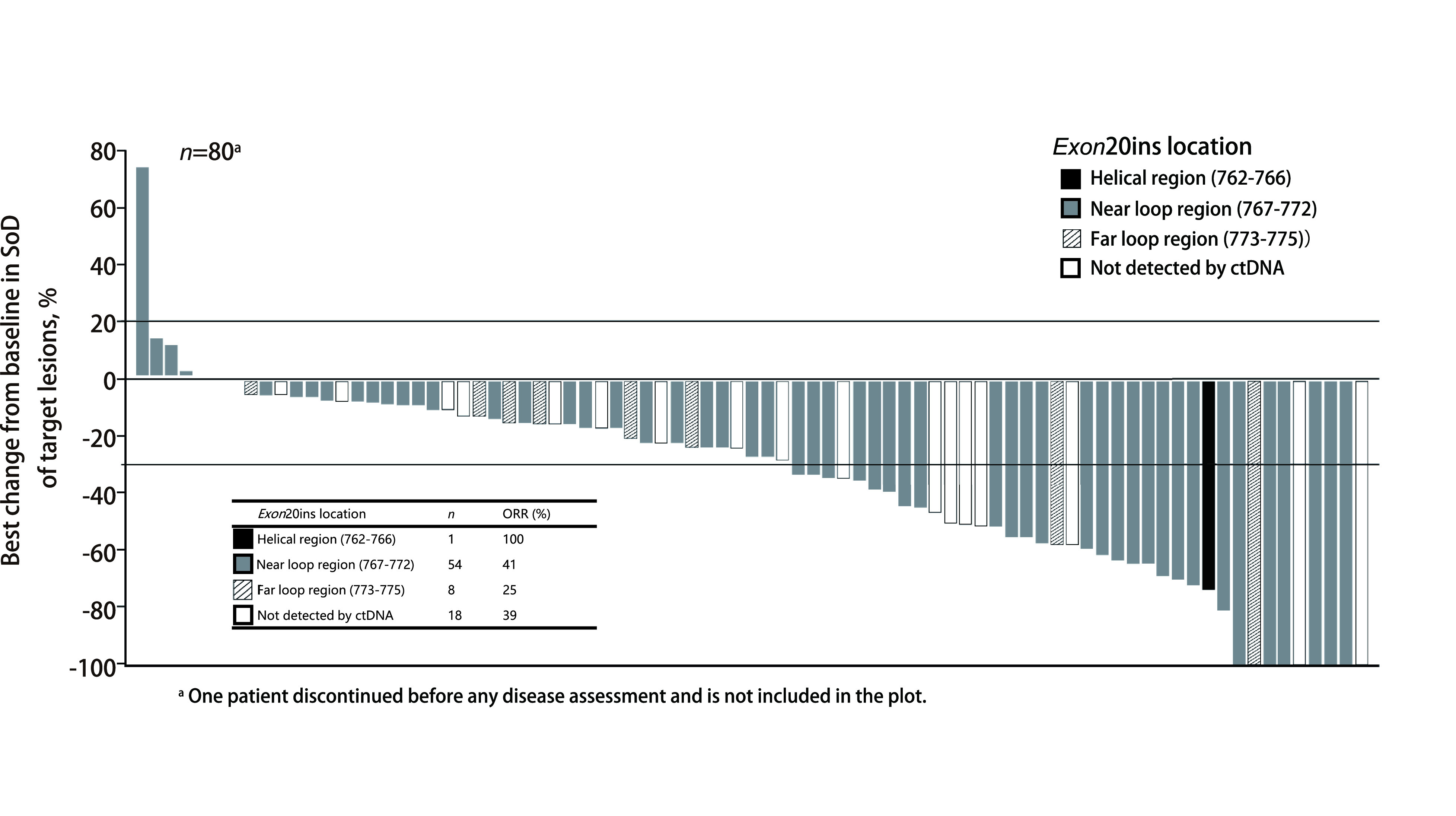
CHRYSTALIS有效性人群中不同*EGFR*外显子20插入突变位点与插入结构区亚组的肿瘤缩小与缓解情况^[47]^。 Tumor reduction and responses by insert site of *EGFR* exon 20 insertion mutation in efficacy population of CHRYSTALIS^[47]^. SoD: sum of lesion diameters; ctDNA: circulating tumor DNA.

注：本图得到版权所有者© 2021 by American Society of Clinical Oncology复制许可。

Note: Reprinted with the permission from the copyright holder © 2021 by American Society of Clinical Oncology.

安全性数据集共纳入114例患者。安全性特征与EGFR和MET通路抑制剂一致。几乎全部患者出现治疗相关的任何AE，前三位分别为皮疹（86%）、输液反应（infusion-related reaction, IRR）（66%）、甲沟炎（45%）。≥3级治疗相关的任何AE发生率为16%，导致减量的AE发生率为13%，导致中断治疗的AE发生率为21%，导致停药的AE发生率为4%。94%的输液反应出现在首次输注，而后续治疗很少发生IRR。此外，MET相关AE包括低白蛋白血症（27%，其中3级为3%）和外周水肿（18%，其中3级为0%）^[47]^。对于输液反应，首次出现指征时需暂停输注。1级-2级情况下，可调整剂量，并在下一次输注前给予皮质类固醇预处理。当出现3级-4级反应时，根据严重程度减缓输注速度或永久性停药^[45]^。

多项Amivantamab研究正在积极开展中。全球多中心、随机、开放性、Ⅲ期研究PAPILLON（NCT04538664）将评估Amivantamab联合卡铂与培美曲塞*vs*卡铂与培美曲塞一线治疗*EGFR*外显子20插入突变阳性的局部晚期或转移性NSCLC的安全性和有效性^[48]^。该研究允许交叉给药，有助于为单药Amivantamab能否最终取代化疗的一线地位提供线索。CHRYSALIS-2（NCT04077463）将探索Amivantamab联合Lazertinib用于携带*EGFR*突变的经治NSCLC，其中包括外显子20插入突变队列。该研究设计的理论基础建立在CHRYSALIS剂量扩增组数据之上，这一联合方案在奥希替尼治疗复发后的患者中显示了有效性^[49]^。

### 3.3 热休克蛋白90（heat shock protein, Hsp90）抑制剂

Hsp90抑制剂Luminespib（NVP-AUY922）抑制外显子20插入突变共选择的Hsp90伴侣系统，以防止泛素介导的蛋白降解。

Luminespib在*EGFR*外显子20插入突变阳性Ⅳ期NSCLC中的Ⅱ期研究（NCT01854034）中，入组的患者既往治疗线数中位值为1（1-5）。5例患者达到PR，ORR为17%（5/29）；中位PFS为3.3个月，中位OS为12.8个月（*n*=27）。最常见的药物相关AE为腹泻（任何等级83%，无3级）、眼毒性反应（任何等级76%，3级3%）。所有3级AE均为可逆的。59%的患者因药物相关AE需中断或推迟给药^[50]^。

### 3.4 其他药物

其他处于研发早期阶段的药物包括：①Tarloxotinib：为缺氧激活前体药物，能够在病理生理缺氧情况下释放Tarloxotinib-E，后者可能对大多数*EGFR*外显子20插入突变阳性NSCLC有疗效，包括奥希替尼前线治疗失败的患者。耐药机制受到基线期*EGFR*外显子突变亚型的影响，T790M或C797S可导致对Tarloxotinib-E耐药^[51]^；②JS 111（AP-L1898）：为EGFR-TKI，Ⅰ期/Ⅱ期研究（NCT04993391）将评估JS 111在携带*EGFR*外显子20插入突变或其他罕见*EGFR*突变且不适合根治性化放疗的局部晚期或转移性NSCLC中的安全性、耐受性、药物代谢动力学特征以及有效性；③BDTX-189：为*EGFR*与*HER2*抑制剂，MasterKey-01临床前模型预测人体内活性剂量为400 mg-800 mg，每日1次。该研究仍在进行中，包括改良剂量方案和确定推荐的Ⅱ期剂量等^[52]^；④吡咯替尼：为泛HER-TKI，Ⅱ期研究（NCT04063462）将评估吡咯替尼在携带*EGFR*或*HER2*外显子20插入突变且既往接受过≥一线治疗的局部晚期或转移性NSCLC中的缓解率等；⑤JMT101：为全人源单抗。Ib期研究（NCT04448379）将评估JMT101联合阿法替尼或奥希替尼在携带*EGFR*外显子20插入突变的局部晚期或转移性NSCLC且既往一线治疗失败或初治患者中的有效性和安全性；⑥Alflutinib（AST2818/Furmonertinib）：为第三代EGFR-TKI。Ib期研究FAVOUR（NCT04858958）将评估不同剂量Furmonertinib在*EGFR*外显子20插入突变阳性的局部晚期或转移性NSCLC中的初步有效性和安全性，初步结果显示出良好的抗肿瘤活性和耐受性^[53]^；⑦BLU-451：为共价小分子*EGFR*外显子20插入突变抑制剂，能够渗透中枢神经系统，将在携带*EGFR*外显子20插入突变肿瘤的Ⅱ期研究（NCT05241873）中进行评估（表 2）。

**表 2:** 针对EGFR外显子20插入突变阳性NSCLC的药物研发进展^[51-53]^ Research and development of drugs for EGFR exon 20 insertion mutation positive NSCLC^[51-53]^

Drug	MOA	Manufacturer	Study	EGFR ex20ins mutation positive NSCLC included	Estimated enrollment	Efficacy	Safety profiles	Others
Tarloxotinib^[51]^ (Hypoxia activated prodrug pan-HER2 TKI)	Releases a potent and irreversible pan-ERBB TKI (Tarloxotinib-E, the activated form) under pathophysiological hypoxia	Rain Therapeutics	Preclinical research	Transfected Ba/F3 cells	-	Tarloxotinib-E was efficacious against all tested Ba/F3 cells except for H773insH	-	-
JS 111 (EGFR-TKI)	Inhibits uncommon *EGFR* mutations	Shanghai Junshi BioSciences	Open label, single arm, phase 1/2 study (NCT04993391)	Confirmed locally advanced or metastatic NSCLC harboring *EGFR* ex20ins and other rare *EGFR* mutations that cannot undergo radical chemoradiotherapy	156	ORR^*^	Safety^*^ (AEs, SAEs, *etc*)	-
BDTX-189^[52]^ (EGFR and HER2 inhibitor)	Has potent, selective, irreversible inhibition against 48 allosteric *EGFR* and *HER2* mutant variants	Black Diamond Therapeutics	Phase 1 MasterKey-01 (NCT04209465)	EGFR exon 20 insertion PDX in mice	-	-	-	Rapid oral absorption and a short PK T_1/2_; MasterKey-01 is ongoing, including refinement of the dosing regimen and identification of the RP2D and schedule
Pyrotinib (EGFR, HER2 and HER4 inhibitor)	Inhibits the downstream signals of the Akt/p-65/FOXC1 pathway	Hengrui Medicine	Open label, single arm, phase 2 study (NCT04063462)	EGFR or HER2 ex20ins mutation positive advanced NSCLC pts, who failed ≥1 prior therapies	60	ORR^*^	Safety and tolerability†	-
JMT101 (fully human EGFR mAb)	Inhibits EGFR mediated signaling and the proliferation of tumor cells expressing EGFR	China Shijiazhuang Pharmaceutical Group	Open label, multi-center phase 1b study (NCT04448379）	Locally advanced or metastatic NSCLC harboring an EGFR ex20ins mutation; No previous treatment or first-line treatment failed	48	ORR, DCR, PFS, OS^*^	AEs^*^	-
Alflutinib or AST2818/Furmonertinib^[53]^ (third generation EGFR-TKI）	Irreversibly inhibits EGFR autophosphorylation and downstream signaling	Allist Pharmaceuticals	Multi-center phase 1b FAVOUR (NCT04858958)	Locally advanced or metastatic non-squamous NSCLC; local lab confirmed *EGFR* 20ins mutation	30 (20 previously systemically treated and 10 treatment naïve)	Cohort 1 (240 mg *QD*, 10 treatment naïve pts): ORR 60% (IRC), 70% (INV); DCR 100% (IRC and INV)	9 of 10 (90%) pts experienced TEAEs of any grade; AEs were manageable and reversible; no grade ≥3 TEAEs was observed; no incidence of dose reduction or treatment discontinuation due to AEs	Cohort 2 (240 mg *QD*, 10 pre-treated pts) and cohort 3 (160 mg *QD*, 10 pre-treated pts) is ongoing
BLU-451 (EGFR-TKI)	Highly selectively targets *EGFR* exon 20 insertion mutations with brain-penetration	Blueprint Medicines	Phase 2, open-label study (NCT05241873)	Advanced cancers with *EGFR* exon 20 insertion mutations	Estimated enrollment: 96	ORR, DoR, DCR, PFS, OS^*^	AEs_†_	DLT，MTD and RP2D_†_
^*^Primary outcome measure; _†_Secondary outcome measure. DLT: dose limiting toxicity; DoR: duration of remission; mAb: monoclonal antibody; MTD: maximum tolerated dose; OS: overall survival; PDX: human tumor xenograft model; PK: pharmacokinetics; SAE: serious adverse event.								

## 4 当前指南推荐

美国国立综合癌症网络（National Comprehensive Cancer Network, NCCN）与美国临床肿瘤学会（American Society of Clinical Oncology, ASCO）总体意见原则上一致，对于*EGFR*外显子20插入突变阳性的晚期或转移性NSCLC，推荐起始治疗参考无驱动基因的NSCLC系统性治疗策略，即含铂两联化疗±贝伐珠单抗，后续治疗为Mobocertinib或Amivantamab。鉴于Amivantamab和Mobocertinib尚未在中国上市，2021年中国临床肿瘤学会（Chinese Society of Clinical Oncology, CSCO）推荐一线参考无驱动基因的NSCLC一线治疗；进展后，推荐参考无驱动基因的NSCLC的后线治疗（Amivantamab于2021年5月经FDA获批，作为3类推荐），此外，指南注释中也列出了Mobocertinib（2021年9月在FDA获批，2021版CSCO指南更新仅纳入2021年6月前在FDA获批的药物）的Ⅰ期/Ⅱ期研究结果^[8, 9, 54]^。

此外，NCCN指南推荐起始、维持以及后续治疗均可包含免疫治疗，而ASCO指南认为一线治疗不应单用免疫治疗，而免疫治疗联合化疗和/或贝伐珠单抗带来的额外获益仍不明确^[8, 65]^。

欧洲肿瘤内科学会（European Society for Medical Oncology, ESMO）未针对*EGFR*外显子20插入突变的NSCLC进行用药推荐^[55]^。

## 5 展望和结论

*EGFR*外显子20插入突变NSCLC异质性高、预后差、对传统治疗不敏感，未满足的临床需求很高。近年来，*EGFR*外显子20插入突变阳性NSCLC靶向治疗出现了里程碑式的进步，Amivantamab为首个针对这种突变的EGFR/MET双特异性抗体，Mobocertinib为首个特异性设计的新型EGFR-TKI，二者先后获得美国FDA批准以及NCCN指南推荐。两种药物均在样本量相对较大（Mobocertinib研究*N*=114，Amivantamab研究*N*=81）的*EGFR*外显子20插入突变阳性NSCLC中证实带来临床意义上的持续缓解，抗肿瘤活性见于全部亚组与不同插入部位的突变亚型。Mobocertinib与Amivantamab在药物机制与给药途径方面均不同，这为患者创造了更多可能的治疗选择。

针对*EGFR*外显子20插入突变阳性NSCLC的其他药物也有望为患者创造更多的治疗机会，但当前研究样本量都比较小，对结果的解读需要谨慎。需要指出的是，波奇替尼先前研究结果令人鼓舞，但之后研究数据不佳，推测可能归于该药AE发生率较高，因而影响了治疗依从性，该药减量方案的有效性结果值得期待。

对于*EGFR*外显子20插入突变阳性NSCLC，当前临床实践或临床研究背景下的治疗手段仍有相当大的进步空间。*EGFR*外显子20插入尚未纳入常规筛查范围，这可能导致诊断不确切与病例漏报，进而难以达到针对驱动基因阳性的精准治疗。临床医生应在完善的实验室条件下为新确诊NSCLC患者进行生物标志物检测，在可及范围内为患者制定最可能获益的治疗策略，包括参与临床研究。
